# Significant air embolism: A possibility even with collapsible intravenous fluid containers when used with rapid infuser system

**DOI:** 10.4103/0019-5049.60498

**Published:** 2010

**Authors:** Deepanjali Pant, Krishan Kumar Narani, Jayashree Sood

**Affiliations:** Department of Anaesthesiology, Pain and Perioperative Medicine, Sir Ganga Ram Hospital, Old Rajinder Nagar, New Delhi - 110 060, India

**Keywords:** Collapsible plastic bag, rapid infuser system, venous air embolism

## Abstract

Significant venous air embolism may develop acutely during the perioperative period due to a number of causes such as during head and neck surgery, spinal surgery, improper central venous and haemodialysis catheter handling, etc. The current trend of using self collapsible intravenous (IV) infusion bags instead of the conventional glass or plastic bottles has several advantages, one of thaem being protection against air embolism. We present a 56-year-old man undergoing kidney transplantation, who developed a near fatal venous air embolism during volume resuscitation with normal saline in collapsible IV bags used with rapid infuser system. To our knowledge, this problem with collapsible infusion bags has not been reported earlier.

## INTRODUCTION

Air embolism can occur whenever a pressure gradient favouring entry of gas into blood circulation exits. This may happen in case of an injury to the veins above the heart, as in head and neck surgery, or during invasive intervention such as central venous catheterization, tubing changes, etc.[1]

A small amount of air often gets into the blood circulation, which is stopped at the lungs and very rarely produces symptoms. Death may occur if a large bubble of gas becomes lodged in the heart stopping blood from flowing from the right ventricle to the lungs. Although the maximum safe amount of air is unknown, as little as 20 ml/sec of air may show symptoms and 70-150 ml/sec of air can be fatal.[2]

Now, the glass bottles for IV fluids have been replaced by plastic containers for several advantages like ease of handling, use with rapid infuser system. Moreover, the self collapsible plastic IV infusion bags are preferred over conventional plastic containers for added margin of safety against inadvertent air embolism.

We report a case involving a near fatal air embolism through the central venous catheter during the volume resuscitation phase during kidney transplantation with the help of pressure infuser bag over the self collapsible plastic bags. Recommendations to prevent such a catastrophic occurrence are also given.

## CASE REPORT

A 56-year-old male weighing 60 kg was scheduled for live related allograft kidney transplantation. He was diagnosed to have end-stage renal disease and was on regular bi-weekly maintenance haemodialysis. The patient was on regular medications like antihypertensive drugs, calcium and phosphorous supplements. He had an adequate cardio-respiratory reserve with a normal chest X-ray, ECG and resting 2-D echocardiogram. All investigations, except an Hb level of 8.5 gm% and serum creatinine of 5.8 mg/dl, were within normal limits.

The patient was haemodialysed the previous day and premedicated with tablet alprazolam 0.25 mg orally the night before the operation and on the morning of surgery. The morning doses of antihypertensive drugs were also given with a few sips of water. His preoperative pulse rate, BP, RR, SpO_2_ were 80/min, 140/80 mm of Hg, 14/min and 98% respectively.

General anaesthesia was induced with fentanyl 100 μg, midazolam 1 mg, thiopentone 250 mg and atracurium 30 mg IV. Trachea was intubated with cuffed ETT of 8.5 mm ID and anaesthesia was maintained with O_2_: air (40:60), isoflurane 0.6-1% and atracurium infusion @ 0.5 mg/kg/hr. For invasive monitoring triple lumen CVP catheter was placed via the right internal jugular vein approach and right radial artery was cannulated. The surgery was proceeding uneventfully with continuous monitoring of pulse, SpO_2_ EtCO_2,_ ECG, ST analysis, temperature, IBP, CVP and intermittent ABG analysis (which were all within normal limits). Vascular anastomoses were being done after clamping the external iliac artery and vein. The base line CVP of 8 mm Hg was raised up to 20 mm Hg by rapid infusion of normal saline (collapsible IV bags) with the help of pressure infuser bags and simultaneous 20% albumin infusion. This was done to achieve supranormal intravascular volume to ensure adequate perfusion to the new renal graft. Just after unclamping of the vascular clamps there was a sudden decrease in EtCO_2_ from 32mm Hg to 15mm Hg followed by decrease in the SpO_2_ from 98 to 66% with significant bradycardia (HR <45/min) and hypotension. Since there was a clinical suspicion of sudden air embolism (most probably due to entrainment of air in the IVC), immediate resuscitative measures including flooding of operative site with saline, head down with left lateral tilt (Durrant's position), IV atropine 0.6 mg, ventilation with 100% O_2_ and aspiration of air through distal lumen of CVP line were tried. No obvious air was seen on aspiration and SpO_2_ returned to 100% with normal EtCO_2_ tracing within a minute.

The surgery proceeded uneventfully with an adequate urine output. The trachea was extubated on the operating table and the patient was shifted to kidney transplant ICU for observation and further management. However, the surgeon was doubtful about entrainment of air into IVC as there was no leak around the anastomotic site. Subsequently we observed that one of the IV sets connected to the proximal lumen of CVP line had air in it and the IV fluid bag inside the pressure infuser bag was empty.

On retrospective analysis, it was realized that the self collapsible one litre NS bag was disconnected during the infusion and IV mannitol was connected in that line. Later, after the mannitol infusion, the same NS bag was reconnected and the pressure infuser bag was applied to it for the rapid infusion which resulted in air embolism as the saline infusion had finished unnoticed and the pressurized air entered through the central venous catheter leading to sudden fall in EtCO_2_, HR, BP and SpO_2_.

As this life threatening situation was immediately recognized due to standard monitoring protocols, the resuscitation measures were started. Left lateral tilt might have allowed the air bubble obstructing the RV outflow tract to be shifted to the superiorly placed right atrium, thereby, allowing the oxygenation and circulation to be restored. The drop in saturation level could have been attributed to overloading with IV fluids, but it would not have been as sudden as in our case. Moreover, the SpO_2_ in Durrant's position would have worsened in case of fluid overload. Hence fluid overload was unlikely in our case. We established this as the possible cause by simulating the same scenario *in vitro*.

## DISCUSSION

When air enters the veins, it travels to the right heart and then the lungs. This can cause the pulmonary vessels to constrict raising the pressure in right heart. If the pressure rises high enough in a patient with patent foramen ovale (present in 10-35% of population), the gas bubble can travel to the left side of the heart and on to the brain or coronary arteries which are responsible for the most serious of gas embolic symptoms. A relatively large volume of air (5-8 ml/kg body wt) can be tolerated in the RV and pulmonary artery, whereas, as little as 0.5 ml of air can be lethal when entered into the left side of circulation.[3,4]

Clinical presentation of right sided air emboli include shock or electromechanical dissociation (EMD) whereas left sided air embolism is evident through its effects on coronary circulation i.e. arrhythmia (especially ventricular ectopy, asystole or EMD) or effects on cerebral circulation (dizziness, loss of consciousness, seizures, blindness, paresis etc).[5,6]

Administrating IV fluids involving glass containers suffer from several disadvantages like difficult to handle, inability to use in rapid infuser bag and risk of leaching. The current trend of using plastic infusion bags has many advantages over glass containers, but also has a few demerits. The ideal plastic container should be substantially chemically inert, nonbreakable, light-weight, exceedingly compact, sterilisable at 121° Celsius, self- collapsible, transparent, equipped with closed infusion system, multilayer protection with higher adaptability and flexibility.

Self-collapsible nature allows proper utilization of fluid, easy monitoring of flow and protects against air entrapment. The closed infusion system does not require an air vent, thereby protects against nosocomial infection and also has a dedicated extra medication port having leach free rubber with self-sealing property.

The self-collapsible bags, particularly those without self-sealing outlets, should not be disconnected from the IV set before completion. The self collapsible nature itself is a safety measure to prevent air embolism even if it finishes unnoticed. But once it is disconnected in the middle of the infusion, the air which gets into the bag can cause air embolism when given with a pressure infuser system unless carefully noticed and closed before the bag is empty. In such a situation the outlet of the bag may be clamped by an artery forceps or plugged with a plastic cover of the hypodermic disposable needle. It would be ideal to remove the container from IV stand and turn it to an upright position. Then air should be evacuated by squeezing the bag from below in upright position and then insert the IV set to continue administration. This problem is not seen with collapsible bag with self-sealing outlet (providing closed infusion system).

### Recommendations to prevent air embolism

- Vigilance and use of proper monitoring equipment
- Use of luer-locks to avoid CVP line disconnection
- Use of check valves to prevent entrainment of air even after disconnection
- Use of an air-in-line detector with infusion devices

The optimal management for this kind of iatrogenic complication is prevention. However, a high index of suspicion with prompt and aggressive management can save a fatal outcome.

## CONCLUSION

Air embolism is not a problem when the currently available collapsible IV containers are used in accordance with the manufacturer's instructions as closed infusion system.

A single air bubble in a vein does not stop the heart as it is very small. However, such accidentally introduced bubbles may occasionally reach the arterial system through a patent foramen ovale and can cause random ischaemic damage, depending on their route of arterial travel.

This case report intends to suggest the possibility of life threatening air embolism even with self-collapsible bags (which itself is a safe guard to prevent air embolism) when used with rapid infuser system. Self-collapsible bags with self-sealing outlets are comparatively expensive and not widely available. Thus, in common day-to-day practice, when these self-collapsible plastic bags without self-sealing outlets are disconnected from the infusion set once and reconnected for continuation of infusion, air embolism remains a possibility. So we emphasize the importance of being vigilant and different ways to prevent air embolism.
